# Psychosocial factors at work in industries in the countryside of São Paulo

**DOI:** 10.47626/1679-4435-2025-1426

**Published:** 2025-08-25

**Authors:** Maíra de Cazeto Lopes, Sergio Roberto de Lucca

**Affiliations:** 1 Departamento de Saúde Coletiva, Universidade Estadual de Campinas, Campinas, SP, Brazil

**Keywords:** occupational stress, work, organizations, estresse ocupacional, trabalho, organizações

## Abstract

**Introduction:**

The literature highlights psychosocial factors at work as major causes of increased occupational stress. These factors are also associated with musculoskeletal disorders and mental and behavioral issues among workers. Objectives: To describe and analyze the relationships between management characteristics and psychosocial factors at work, as perceived by industrial workers, and their impact on increased work-related stress.

**Methods:**

This is a case study with a qualitative approach, using in-depth interviews and participant observation during fieldwork.

**Results:**

The 5 companies studied predominantly employed a Taylorist production model. Rigid hierarchical management was observed, as well as encouragement of competitiveness among workers, poorly trained leadership, and insufficient investment in training and Occupational Health and Safety. Additionally, there was a disregard for psychosocial factors that can trigger stress and illness.

**Conclusions:**

Our results demonstrated that, irrespective of the specific industrial activity, the primary psychosocial factors at work were overwork, lack of autonomy and control over tasks, lack of social support, lack of career planning and recognition, poor communication, and psychological abuse, including moral harassment. The findings suggest that organizational management should focus on actions to prevent psychosocial factors at work in order to promote workers’ well-being.

## Abstract (pt)

**Introdução:**

Fatores psicossociais no trabalho são apontados na literatura como uma das principais causas do aumento do estresse ocupacional, além de estarem associados à ocorrência de distúrbios osteomusculares e transtornos mentais e comportamentais entre trabalhadores.

**Objetivos:**

Descrever e analisar as relações entre características da gestão e fatores psicossociais no trabalho na percepção de trabalhadores da indústria, investigando seu impacto no aumento do estresse laboral.

**Métodos:**

Tratase de um estudo de caso com abordagem qualitativa, utilizando entrevistas em profundidade e observação participante do trabalho em campo.

**Resultados:**

Nas cinco empresas estudadas, o modelo de produção predominante foi o taylorismo. Observou-se uma gestão hierárquica rígida, estímulo à competitividade entre os trabalhadores, lideranças pouco capacitadas, baixo investimento em treinamentos e em Saúde e Segurança no Trabalho, além da negligência em relação a fatores psicossociais que podem desencadear estresse e adoecimento.

**Conclusões:**

Os resultados deste estudo demonstraram que, independentemente do tipo de atividade produtiva do setor industrial, os principais fatores psicossociais no trabalho identificados foram excesso de demandas laborais, falta de autonomia e controle sobre as atividades, ausência de apoio social, inexistência de plano de carreira e reconhecimento, comunicação deficiente, violência psicológica e assédio moral. Os achados sugerem a necessidade de direcionar a gestão organizacional para ações de prevenção dos fatores psicossociais no trabalho, a fim de promover a saúde dos trabalhadores.

## INTRODUCTION

In recent decades, the world of work has experienced major transformations driven by technological advancements and new organizational management practices. The relentless pursuit of increased profitability and productivity has proven to have a detrimental impact on the workforce, leading to increased precarity and exclusion from the formal market, particularly in central and, above all, peripheral capitalist countries.^1,2,3,4^

Until the 1970s, industrial production processes were based on Taylorism/Fordism, characterized by assembly lines and division of intellectual and manual labor, resulting in alienating, repetitive activities devoid of meaning and subjectivity for workers. Globalization and new technologies have increased international competition and required companies to adapt to a more selective, segmented, and flexible market, promoting changes in the production process.^5,6^

Toyotism, organized into production cells and characterized by flexibility and versatility, aimed to maximize productivity through greater worker engagement with the “capitalist values” of organizations.^7,8^

Industry 4.0, driven by technological advancements, has further automated and robotized production processes throughout the value chain, now controlled digitally. This advancement has also contributed to the individualization and invisibility of human labor and labor relations. Results-based management and new work organization methods have improved control mechanisms and increased individual competition, resulting in the intensification of work and the introduction of new cognitive demands.^5^

Since the 1980s, there has been a global rise in musculoskeletal disorders, followed by a surge in work-related mental disorders (WMDs) from the 1990s onwards. These health issues are now major causes of illness and absenteeism, both globally and in Brazil.^9,10^

International agencies emphasize the role of psychosocial factors at work (PFWs) in causing occupational stress and contributing to musculoskeletal, mental, and behavioral disorders. These PFWs stem from the interplay between workers’ expectations, capabilities, needs, and culture and the organizational demands of work processes and productivity.^9^

PFWs include work content, workload and pace, working hours and periods, degree of control, environment and equipment, organizational culture, role performed, interpersonal relationships in the workplace, organizational role, career development, and the work-home interface.^11^

In 2022, the World Health Organization (WHO) published guidelines on mental health at work, recommending actions such as collective assessment, manager and worker training, and the management and mitigation of work-related psychosocial risk factors. These guidelines emphasize the importance of participation and training at all hierarchical levels to ensure a healthy and safe work environment.^12^

Currently, the industrial production sector employs approximately 22% of the global workforce, and 19% of the formal workforce in Brazil. In the state of São Paulo, the industrial sector leads in absences due to typical accidents and work-related illnesses, according to the National Classification of Economic Activities (Classificação Nacional de Atividades Econômicas, CNAE).^10,13^

Given the limited research on work processes, management models, and illness triggers among industrial workers, the objective of this study was to describe and analyze the relationships between management characteristics and PFWs, as perceived by industrial workers, and their potential contribution to increased work-related stress.

## METHODS

This is a case study with a qualitative approach, using in-depth interviews and participant observation during fieldwork.

The research was conducted across 5 factories located in the regions of Indaiatuba and Sorocaba, in the countryside of the state of São Paulo. Between May and July 2024, the companies were visited to gain insight into their work processes, management methods, and the primary PFWs present in these organizations. Informal conversations with workers, observations of work processes, and notations on workers’ body language and facial expressions while performing their tasks were recorded in a field notebook and subsequently used to develop the interview scripts.

The semi-structured interview format, using both closed- and open-ended questions, offers researchers greater control over the investigation’s direction while providing interviewees space for free and spontaneous reflection.^14^ Interview topics included length of service at the company changes in function and management, work demands, productivity and goal-related pressures, turnover, and the perceived influence of PFWs and organizational aspects on job dissatisfaction and increased stress.

The eligibility criteria for participation in the study included employment at the company for more than 1 year, provision of written informed consent, and authorization for the interview to be recorded. To ensure a representative sample, we considered the workers’ average length of employment at the company and selected 3 to 4 participants from each company using the saturation criterion. Thus, in-depth interviews were conducted with 17 workers of both sexes from 5 companies: 2 from the metallurgical sector (employing 230 and 175 workers each), 2 from the chemical sector (employing 305 and 151 workers each), and 1 from the food sector (employing 73 workers).

All interviews were conducted during working hours, in a location reserved by the companies, and each lasted 20 to 40 minutes. The interviews were recorded and transcribed. To ensure participant anonymity, narratives were coded by company (C1, C2, and so on).

The project was approved by the Research Ethics Committee of Universidade Estadual de Campinas (UNICAMP), under opinion number 5,854,871. All participants provided written informed consent after being informed of the research objectives and the measures taken to preserve their identities.

## RESULTS

Among the sample participants, the average length of service at the company was 4 years, and half were promoted from production assistants to operators.

Chart 1 describes the 5 companies in the sample, categorized by their business activities, the origin of their economic capital, and similarities in organizational culture and management methods. The predominance of Taylorism was observed in production, characterized by hierarchical and rigid management that stimulates competitiveness among workers and features poorly trained leaders. The Occupational Health and Safety (OHS) sector is represented by an occupational safety technician and outsourced OHS companies, primarily focused on regulatory compliance.

**Chart 1:** Characteristics associated with business activities and economic capital, production models, work organization, and PFWs identified in the 5 factories located in the regions of Indaiatuba and Sorocaba, in the countryside of the state of São Paulo

Business and capital	Production model	Characteristics of work organization	OHS	PFWs
Chemical; Brazilian Metallurgical; Brazilian Metallurgical; Japanese Food; French	Taylorism	Rigid hierarchy; poorly qualified leadership; focus on productivity and product quality; encouragement of competitiveness.	Occupational safety technician responsible for OHS; insufficient training; low investment in environment and equipment; outsourced occupational medicine to meet legal demands (medical examinations); and specific cases of absences and restrictions resulting from occupational accidents and diseases.	Lack of autonomy and control; lack of social support; limited participation in decision-making; poor communication between workers and leaders; heavy workload; lack of career planning and recognition; psychological abuse and moral harassment.
Chemical; Japanese	Toyotism/Taylorism			

PFWs = psychosocial factors at work ; OHS = Occupational Health and Safety.

Regarding the organizational characteristics of the companies, one of them, which operates with national capital, exhibits a paternalistic management style characteristic of family-owned businesses. Within this organization, there is a prevailing view of the workforce as subservient and working for survival. Investment in technology is low, and leadership selection lacks professionalism. Production adheres to Taylorist principles, resulting in a predominance of manual and alienated labor. Workers are treated as mere components of the production system, with little autonomy or control over their work. They are expected to be versatile, moving between sectors and jobs without adequate training, yet are held responsible for the quality of their output. During the off-season, the owners request other tasks that involve job deviation, causing them discomfort.

In the 2 metallurgical companies, a rigid and well-defined hierarchy was observed, with leadership hired externally and possessing limited experience in the production process. In both organizations, the work process is predominantly Taylorist in nature, with low investment in technology. Production goals are clearly defined, yet the intense work pace and high work demands often lead to overtime and difficulties in meeting these goals. Poor interpersonal relationships were also observed, characterized by a lack of support from coworkers and managers and the presence of management practices that could be construed as harassment.

In one of the Japanese-owned companies, while the organization maintains a focus on excellence, its production methods remain rooted in traditional assembly lines, with strictly defined work processes and clear production goals. The company adopts rest breaks and task rotations. Investments in technology and ergonomics are evident. However, there is strong emphasis on time control, with clocks and stopwatches attached to machines and workstations. Quality control is closely monitored, with each product bearing a label that identifies the responsible worker. Interaction between workers is low, and a sense of competition is fostered by management. The company offers a career progression plan, with opportunities to advance into leadership roles. The board of directors, comprised solely of Japanese individuals, maintains a strong and active presence on the shop floor and is rotated every 2 years.

The French-owned company operates with a paternalistic management style and a rigid hierarchical structure. Its production model follows Taylorism, with workers assigned to different tasks based on a daily production schedule. The pace of work and production line speed are determined by management. Limited interaction was observed among coworkers. As a food industry company, the facilities are maintained in a clean and organized manner, adhering to strict quality control requirements. The leaders are well-trained and attain their positions through career progression or direct hiring. Recently, the company implemented workforce reductions, leading to increased work demands for the remaining employees.

After floating reading the content and analyzing the recorded interviews with 17 production line workers and 7 managers, we developed the thematic matrix “psychosocial and organizational factors at work” and identified 4 analytical categories: autonomy and control; support from management and coworkers; recognition; and abuse and moral harassment.

## DISCUSSION

Work organizations are shaped by the origin of controlling capital and the political and economic contexts in which they are inserted. These influences introduce cultural, value-based, social class, and ethnic differences that affect labor relations, career paths, and various situations within the workplace, such as PFWs.^5,15^ Management practices, particularly those with rigid rules that disregard the socio-historical context and worker expectations, can lead to alienation and dissatisfaction related to the meaning and experience of work.^16^

Based on the results displayed in Chart 1, the companies under study varied in terms of their business activities and capital origins. The Taylorist production model predominated in these companies. However, one company exhibited similarities to Toyotist management and work organization, with a focus on excellence, productivity, and efficient worker performance. Investment in OSH was low, primarily driven by legal compliance.

Regardless of the type of activity or production model, the main PFWs identified were overwork, lack of autonomy and control, lack of social support, limited participation in decision-making, poor communication, lack of career planning and recognition, and psychological abuse, including moral harassment.

Managerialism based on quantitative productivity metrics, such as the Taylorist production system, neglects the creative potential of workers and inhibits their ability to express their subjectivity in their work. Repetitive and alienating work can lead to adverse health effects.^16^ Work-related distress and illness are often responses to the constraints imposed by the work organization, especially the demand for increasingly challenging individual performance goals.^17^

The individual pressure to meet performance goals and productivity targets intensifies the pace of work and increases psychological demands. When combined with a lack of control and autonomy over tasks, this can severely impact mental health, elevate stress levels, and contribute to physical and mental illness among workers.^18^

In this context, studies have shown that management practices, work organization, and PFWs are associated with heightened stress, suffering, and physical and mental illness. These can manifest in various health issues, including psychiatric disorders, immune system dysfunction, strokes, acute myocardial infarctions, gastric ulcers, and suicides.^19,20,21,22^

Our study found that a lack of autonomy and control over work activities was a major stressor for the interviewed workers, according to their statements: C2: *“I dont have the autonomy to do my work... I cant solve a problem without asking my leader... I feel useless”)* and C4: *“If the machine stops, the clock also stops ticking and we have to justify ourselves, even if it’s to go to the restroom... This is a way of controlling us, which only shows how much they dont trust us”*

The lack of social support from both leaders — in problem solving and personal development — and coworkers — in providing solidarity to carry out tasks — poses a potential risk for illness and can cause psychological and physical harm to workers.^18^ This is highlighted in their statements: C1: *“I don t feel comfortable bringing up problems, because they dont listen to our opinion, only theirs counts.”;* and C5: *“I don’t feel like I have support from my leader, he makes a difference between me and the other workers.”*

Social support by management and coworkers increases employees’ sense of well-being, while a lack of support can trigger work-related stress and diminish quality of life at the workplace. This can lead to burnout and mental health issues, such as depression, anxiety, and post-traumatic stress disorder.^22,23^

The perception of inadequate reward for efforts invested in work, whether symbolic or financial, can heighten stress and negatively affect the physical and mental health of workers.^24^ Recognition plays a crucial role in shaping an individual’s work identity and can transform psychological distress into satisfaction. Conversely, a lack of recognition can lead to feelings of injustice, frustration, and devaluation, potentially resulting in psychological illness.^25,26^

Lack of recognition can contribute to job dissatisfaction, as reported by the interviewed workers: C2: *“I have been in this position since I joined the company and my salary never increases-, I earn the same as my colleague who joined us 2 months ago”; “The company should provide financial recognition as responsibilities increase.”* There are also complaints regarding professional recognition: C4: *“I made a change/improvement to a device... But I feel like I was not recognized for what I did. I felt like crap ...”*

In Brazil, abuse and harassment have been included in the list of work-related psychosocial risk factors and are associated with both physical illnesses (including diseases of the circulatory, digestive, and musculoskeletal systems) and psychological illnesses (such as depression, anxiety, and other mental and behavioral disorders). They have even been linked to suicide.^27^

Psychological abuse and vertical moral harassment perpetrated by leaders can be reproduced by workers, as per the following statements: C3: *“Look at your colleague, he can meet all the goals without difficulty, you will fall behind, you will not be able to reach them if you do not do the work faster-, you are very slow”)* C3: *“He is being fussy; outside the company he goes to the gym, does odd jobs, but here he cannot lift weights; he is getting in our way and overloading us”;* and C5: “... *he has even called my attention during my lunch break; he speaks with gestures and makes me nervous and embarrassed, it seems like he is going to make a lunge at me... “.*

Harassing management can also lead to psychosomatic illnesses and symptoms, as illustrated by the following report: C1: *“I have anxiety symptoms because of all this, lack of sleep, tremors, shortness of breath, during the shift, getting worse at the end. I have dermatitis on my scalp, it forms sores, my hair falls out; I dont feel like eating even after I get home.”*

The current work environment, driven by the pursuit of cost reduction, increased profitability and productivity often fosters a workplace conducive to psychological abuse and harassing practices. Workers are subjected to psychological pressure due to unrealistic productivity demands and unattainable goals. This pressure frequently manifests as humiliation from managers, which is repeated daily, affecting the entire workforce and creating a culture of blame when organizational expectations are not met. The internalization of this guilt can trigger illness in workers who feel personally responsible for not achieving the expected performance.^28,29,30^

Given the emergence of psychosocial factors as triggers of occupational stress and the resulting physical and mental health issues, it is crucial to implement effective and efficient policies and actions to prevent work-related psychosocial risk factors and promote mental health within organizations. The goal is to provide a healthy and welcoming environment, free from threats, that fosters professional growth and dignity for all workers.^12^

As a limitation, although the results of this study align with recent literature on the subject, the research was conducted in 5 industrial companies within a specific region, which may not fully reflect the reality of the broader industrial sector. However, this study contributes original insights given the scarcity of published research on PFWs and organizational factors within the industry context in Brazil.

## CONCLUSIONS

Our results demonstrated that, irrespective of the specific industrial activity, the primary PFWs were overwork, lack of autonomy and control over tasks, lack of social support, lack of career planning and recognition, poor communication, and psychological abuse, including moral harassment.

Therefore, the findings suggest that organizational management practices should focus on the implementation of actions to prevent the identified PFWs in order to promote workers’ well-being.

## References

[1] Pelegrini I, Viana HC, Lacerda GC (2023). Neoliberalismo, superexploração no Brasil
contemporâneo e os desafios da classe trabalhadora. Principios.

[2] Galvão A, Castro B, Krein JD, Teixeira MO (2019). Reforma trabalhista: precarização do trabalho e os desafios
para o sindicalismo. Cad CRH.

[3] Antunes R, Praun L (2015). A sociedade dos adoecimentos no trabalho. Serv Soc Soc.

[4] Antunes R (2014). Desenhando a nova morfologia do trabalho no Brasil. Estud Av.

[5] Antunes R (2020). Uberização, trabalho digital e indústria 4.0.

[6] Hayne LA, Wyse ATS (2018). Análise da evolução da tecnologia: uma
contribuição para o ensino da ciência e tecnologia. Rev Bras Ens Ci Tecnol.

[7] Chanlat JF, Vasconcelos EDJ (1995). Recursos humanos e subjetividade.

[8] Tessarini G, Saltorato P (2018). Impactos da indústria 4.0 na organização do trabalho:
uma revisão sistemática da literatura. Prod Online.

[9] International Labour Organisation (1986). Psychosocial factors at work: recognition and control [Internet].

[10] Brasil, Ministério da Previdência Social (2023). Quantidade de acidentes do trabalho, por situação do registro e
motivo, segundo os 200 códigos da Classificação Internacional de
Doenças - CID-10 mais incidentes, no Brasil [Internet].

[11] Leka S, Cox T, World Health Organization (2008). PRIMAEF: guidance on the European framework for psychosocial risk management: a
resource for employers and worker representatives [Internet].

[12] World Health Organization (2022). WHO guidelines on mental health at work [Internet].

[13] Brasil, Ministério do Trabalho e Emprego (2024). Painel de informações do novo Cadastro Geral de Empregados e
Desempregados (CAGED) [Internet].

[14] Minayo MCS (2012). Análise qualitativa: teoria, passos e fidedignidade. Cien Saude Colet.

[15] Vieira CEC, Santos NCT (2024). Fatores de risco psicossociais relacionados ao trabalho: uma análise
contemporânea. Rev Bras Saude Ocup.

[16] Dejours C (2018). A loucura do trabalho: estudo de psicopatologia do trabalho.

[17] Dejours C (2022). Trabalho vivo II: trabalho e emancipação.

[18] Karasek R (2008). Low social control and physiological deregulation— the
stress–disequilibrium theory, towards a new demand–control model. Scand J Work Environ Health Suppl.

[19] Sousa RM, Cenzi CM, Bortolini J, Terra FS, Valim MD (2023). Common mental disorders, productivity and presenteeism in nursing
workers. Rev Esc Enferm USP.

[20] Moronte EA, Albuquerque GSC (2021). Organização do trabalho e adoecimento dos bancários:
uma revisão de literatura. Saude Debate.

[21] Silva AAB, Araújo EABS, Brito ME (2023). Adoecimento mental e suicídio no trabalho: negação da
condição humana pelo trabalho. Rev Esc Nac Insp Trab.

[22] Silva M, Andolhe R, Lima MP, Moreira LP, Souza TCG, Magnago TSBS (2022). Apoio social em trabalhadores da saúde: tendências das
produções nacionais. Res Soc Dev.

[23] Vig KD, Mason JE, Carleton RN, Asmundson GJG, Anderson GS, Groll D (2020). Mental health and social support among public safety
personnel. Occup Med (Lond).

[24] Siegrist J, Starke D, Chandola T, Godin I, Marmot M, Niedhammer I (2004). The measurement of effort–reward imbalance at work: European
comparisons. Soc Sci Med.

[25] Paparelli R, Almeida TB, Silva DLD, Morgado LP (2019). Adoecimento bancário: construção de estratégias
individuais e coletivas para o enfrentamento do desgaste mental relacionado ao
trabalho. Rev Bras Saude Ocup.

[26] Dejours C (2004). Subjectivity, work and action. Prod.

[27] Brasil, Ministério da Saúde (2023). Portaria GM/MS nº 1.999, de 27 de novembro de 2023 [Internet].

[28] Barreto M, Heloani R (2015). Violência, saúde e trabalho: a intolerância e o
assédio moral nas relações laborais. Serv Soc Soc.

[29] Trindade LL, Schoeninger MD, Borges EMN, Bordignon M, Bauermann KB, Busnello GF (2022). Assédio moral entre trabalhadores brasileiros da
atenção primária e hospitalar em saúde. Acta Paul Enferm.

[30] Grotto-de-Souza J, Pohl HH, Aguiar-Ribeiro D (2022). Mobbing as a source of psychological harm in workers. Rev Bras Med Trab.

